# A review on the biology and properties of adipose tissue macrophages involved in adipose tissue physiological and pathophysiological processes

**DOI:** 10.1186/s12944-020-01342-3

**Published:** 2020-07-09

**Authors:** Yunjia Li, Ke Yun, Runqing Mu

**Affiliations:** 1 The First Clinical Medicine Faculty, China Medical University, Shenyang, 110001 China; 2 Department of Laboratory Medicine, The First Hospital of China Medical University, Shenyang, 110001 China

**Keywords:** Obesity, Adipose tissue macrophages, White adipose tissue, Brown adipose tissue, Beige adipose tissue, Inflammation, Lipid metabolism, Energy metabolism, Metabolic disorders

## Abstract

Obesity exhibits a correlation with metabolic inflammation and endoplasmic reticulum stress, promoting the progression of metabolic disease such as diabetes, hyperlipidemia, hyperuricemia and so on. Adipose tissue macrophages (ATMs) are central players in obesity-associated inflammation and metabolic diseases. Macrophages are involved in lipid and energy metabolism and mitochondrial function in adipocytes. Macrophage polarization is accompanied by metabolic shifting between glycolysis and mitochondrial oxidative phosphorylation. Here, this review focuses on macrophage metabolism linked to functional phenotypes with an emphasis on macrophage polarization in adipose tissue physiological and pathophysiological processes. In particular, the interplay between ATMs and adipocytes in energy metabolism, glycolysis, OXPHOS, iron handing and even interactions with the nervous system have been reviewed. Overall, the understanding of protective and pathogenic roles of ATMs in adipose tissue can potentially provide strategies to prevent and treat obesity-related metabolic disorders.

## Introduction

Adipose tissue can be divided into white adipose tissue (WAT) and brown adipose tissue (BAT); the percentage of WAT is up to 5 to 50% of body weight including subcutaneous adipose tissue (SAT) and visceral adipose tissue (VAT), and the percentage of BAT decreases with age [1]. Adipose tissue is not only the body’s energy reservoir to insulate against the cold and protect vital organs but also an essential endocrine organ, especially white adipose tissue, which is the main source of endocrine signals [2].

Macrophages are heterogeneous, and their phenotype and functions are regulated by the surrounding microenvironment [3]. Classically activated M1 or proinflammatory macrophages produce proinflammatory cytokines such as interleukin-1β (IL-1β), IL-6, IL-12, IL-23, and TNF-α, in response to infection and stress. On the other hand, alternatively activated M2 or anti-inflammatory and immunoregulatory macrophages produce anti-inflammatory cytokines such as IL-10 and TGF-β, contribute to tissue repair, remodeling, and vasculogenesis, and maintain homeostasis [4, 5]. Macrophages exploit protective and pathogenic roles in anti-infection defense, antitumor immunity, metabolic disease development, and even obesity [6].

Adipose tissue macrophages (ATMs) are pivotal players in obesity-associated inflammation and metabolic diseases [7]. Macrophages are key modulators of energy metabolism and mitochondrial function in adipocytes [8]. It seems that ATMs develop from circulating monocytes accumulating in adipose tissue, self-renew from various tissue-resident macrophages [9], or proliferate in situ driven by monocyte chemotactic protein 1 (MCP-1), which is an important process for macrophages accumulating in VAT in obesity [10]. The number of tissue-infiltrating macrophages is higher in superficial adipose tissue than deep adipose tissue, suggesting accessibility to skin microorganisms might promote macrophage infiltration in SAT [11]. Resident ATMs have lower levels of apoptosis and rapid proliferation during early phases of WAT expansion with a high-fat diet (HFD) [12, 13]. Lipid-rich CD11c^+^ ATMs appear earlier in VAT than SAT in response to ectopic lipid accumulation as adipocytes reach maximal lipid storage capacity [13].

**Fig. 1:**
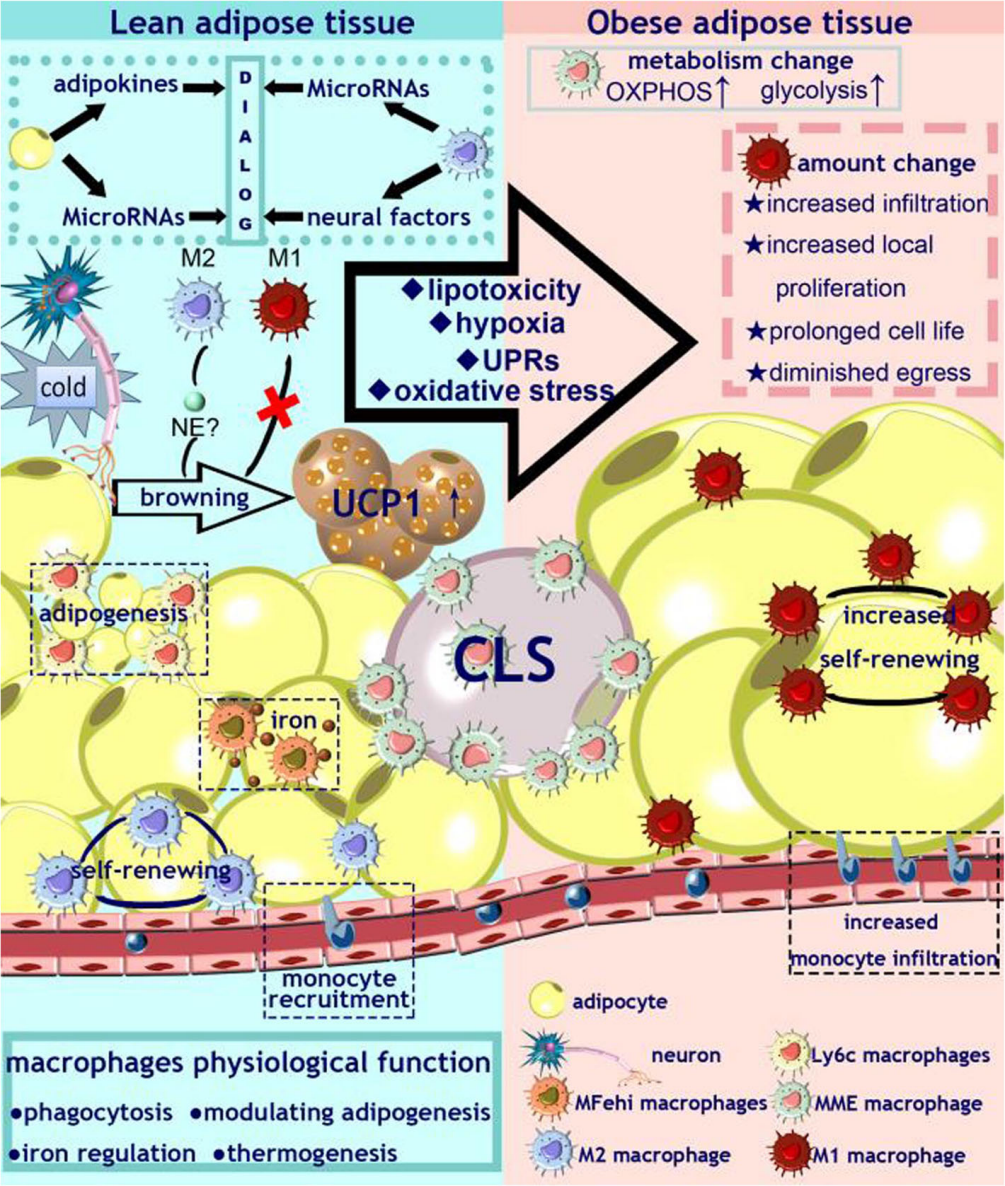
The adipose tissue macrophage (ATM) population is a compound system of embryonic and infiltrating monocyte-derived macrophages with the ability of self-renewal. Under physiological conditions, ATMs with different phenotypes perform a variety of physiological functions. ATMs adopt a metabolically activated (MMe) phenotype to promote dead adipocyte clearance through lysosomal exocytosis. Ly6c ATMs support normal adipose physiology upon adoptive transfer by inducing genes related to cholesterol and lipid biosynthesis. Alternatively activated MFe^hi^ ATMs display elevated cellular iron content along with an anti-inflammatory and iron-recycling gene expression profile. M2 macrophages induce increased UCP1 levels in adipocytes and promote browning by mimicking the sympathetic signaling pathway. The interplay between ATMs and adipocytes in energy metabolism, glycolysis, OXPHOS, iron handing and even interactions with the nervous system. In obesity, the microenvironment of adipose tissue changes dramatically, and lipotoxicity, hypoxia, unfolded protein responses (UPRs), oxidative stress and other harmful pathological changes occur in succession. Under these conditions, ATMs undergo changes in numbers, phenotype, and metabolic state

The quantity and activation state as well as metabolic phenotype of ATMs impact the development of obesity-induced metabolic diseases. Herein, it is reviewed how ATMs are involved in adipose tissue physiological and pathophysiological processes (Fig. 1).

## Macrophage polarization in adipose tissues

### Classically activated M1 macrophage polarization

The classically activated M1 macrophages are critical players in the initiation and maintenance of adipose tissue inflammation and progression of insulin resistance in the whole body. Fatty acids and LPS as obesogenic factors activate macrophage inositol-requiring enzyme 1α (IRE1α), which represses M2 while enhancing M1 polarization. The development of obesity and metabolic syndrome is enhanced by the macrophage IRE1α pathway by impairing BAT activity and WAT browning [14]. Excess glucose directly affects macrophage activation via the ROCK/JNK and ROCK/ERK pathways, which induce human monocytes and macrophages to undergo M1 polarization upon exposure to high levels of glucose [15]. miR-30 is downregulated in HFD-induced obesity via DNA methylation, thereby inducing Notch1 signaling in ATMs and promoting M1 macrophage polarization [16].

Bone-marrow-derived macrophages isolated from Nfatc3^−/−^ mice treated with IFN-γ and lipopolysaccharide resulted in a reduction in M1 inflammatory markers in vitro, suggesting that Nuclear factors of activated T cells (NFAT) c3 promoted M1 polarization in a cell-autonomous way [17]. Fibronectin type III domain-containing protein 5 (FNDC5), a novel myokine secreted by contracting skeletal muscle, can attenuate inflammation and insulin resistance through AMPK-mediated macrophage polarization in HFD-induced obesity [18].

### Alternatively activated M2 macrophage polarization

The alternatively activated M2 macrophages are the predominant macrophage phenotype responsible for anti-inflammation in lean animals. M2 macrophages in adipose tissue inhibited adipocyte progenitor proliferation in the CD206/TGF-β signaling pathway to modulate systemic glucose homeostasis [19]. Deficiency of TLR4 induces the M2-macrophage phenotype and adipose tissue fibrosis [20]. ATMs express NPFFR2, a receptor for the appetite-reducing neuropeptide FF (NPFF), whose plasma levels decrease in obesity, and NPFFR2 deficiency in ATMs abolished both M2 activation and ATM proliferation [21].

It has been indicated that IL-25 stimulates alternatively activated macrophages and their interaction with adipocytes but promotes energy metabolism, enhances mitochondrial functions and attenuates lipid accumulation in the liver and adipose tissues [22]. In addition, cannabinoid receptor 1 (CB1) blockade resulted in downregulation of miR-466 family and miR-762 in ATMs, which promote M2 polarization and macrophage egress from adipose tissue [23]. Empagliflozin, a sodium-glucose cotransporter (SGLT) 2 inhibitor, repressed weight gain by enhancing browning of adipocytes and alleviated obesity-induced inflammation and insulin resistance by polarizing M2 macrophages in WAT and the liver [24]. Similarly, Telmisartan, a well-known antihypertensive drug, was reported to promote the browning of fully differentiated white adipocytes partly through PPAR-mediated M2 polarization [25].

Intriguingly, helminth infection significantly alleviated obesity along with significantly increased Th2/Treg responses and M2 macrophage polarization [26]. Adoptive transfer of helminth-stimulated M2 cells to mice without *H. polygyrus* infection conferred an obvious improvement of HFD-induced obesity and adipose tissue browning [26]. In some cases, an intracellular glucocorticoid reactivating enzyme 11β-HSD1 was found to be in the process of switching ATMs from M2 to mixed M1/M2 polarization [27].

### Adipocytes impact macrophages polarization

Adipocytes exert effects on ATM phenotypes via a variety of mechanisms. HFD upregulates the ER stress pathway downstream component CHOP, a transcription factor C/EBP homologous protein, thereby altering WAT microenvironmental conditions including decreased Th2 cytokine and M1 polarization, resulting in insulin resistance and glucose intolerance [28]. Adipocytes release lipid-laden exosomes (AdExos) that deliver triacylglyceride (TAG) locally to macrophages and are able to induce in vitro differentiation of bone marrow precursors into ATMs [29]. It appears that miR-34a expression is elevated in obesity in part through suppression of the browning activators fibroblast growth factor 21 (FGF21) and SIRT1 to inhibit fat browning [30]. AdExos carried miR-34a into adipose resident macrophages, resulting in repression of the expression of Krüppel-like factor 4 (Klf4) to control M2 polarization [31]. miR-155-bearing adipocyte-derived microvesicles (ADM) can regulate M1 macrophage polarization [32, 33]. However, exosomes derived from adipose-derived stem cells (ADSCs) transactivate argininase-1 to drive M2 macrophage polarization. M2 macrophages further favor the proliferation of ADSC and the browning of adipose tissue by releasing catecholamine, forming a positive feedback loop [34]. The molecular and epigenetic factors that influence macrophages polarization in both physiologic and pathologic wound healing have been reviewed in [35].

## Adipose tissue macrophage subsets with potential functions

Scavenging of adipocyte debris is a crucial function of ATMs in obese individuals. Due to their inability to engulf adipocytes debris in one step, macrophages infiltrate and aggregate in WAT to form a crown-like structure (CLS) that envelopes and ingests the moribund adipocyte at sites of adipocyte death [36]. The tissues are protected from hypoxia and ectopic accumulation from remnant lipid droplet through CLS, which is of extracellular lysosomal compartments [36]. ATMs exert lysosomal activity through two vesicles of different pH. One is a neutral lipid vesicle and the other is an acidic-ringed secondary lysosome involved in lipid catabolism, which is formed by fusion of the first vesicle with the primary lysosome [8]. ATMs localize to CLS with various phenotypes. Moreover, MFe ATMs and antioxidant macrophages (Mox) ATMs are essential to iron and oxidative stress handing, respectively. Furthermore, macrophages polarize in both VAT and subcutaneous abdominal adipose tissue. Hence, multiple ATM phenotypes with potential functions have been reviewed in [Table 1].

**Table 1:** Summary of ATMs phenotypes with potential functions in adipose tissues

	Stimulus	transcription factors	Cell surface markers	Cytokines	Functions
MMe macrophages	High levels of glucose, insulin, and palmitate [37]	p62 PPARγ [37]	ABCA1 CD36 PLIN2 [37]	IL-6 (NOX2-dependent) [38]	Removing dead adipocyte debris [37, 39]
CD9 macrophages		AP-1 subunit JunB NF-κB subunit p65	CD9 CD16 CD206	IL-1α IL-18 TNF	Filled with lipids, and secret exosomes [40]
Ly6c macrophages		CTCF [40, 41]	CD11b Ly6c	Factors that support vascular development and organization	Regulating adipogenesis process
MFe^hi^ macrophages	High iron		CD163 Tfrc Hmox1 ferritin light and heavy chains (Ftl1 and Fth1, respectively) ceruloplasmin(Cp) ferroportin-1(Slc40a1)	IL-10	Iron regulation [42, 43]
Antioxidant macrophages (Mox)			• CX3CR1^neg^ F4/80^lo^HO1^+^Txnrd1 [44]		Predominant ATMs phenotype in lean adipose tissue. Response to oxidized phospholipids (OxPLs) by upregulating Nrf2-dependent antioxidant enzymes [45]. Antioxidant macrophages (Mox) require suppression of regular energy metabolism to produce the antioxidant glutathione [46].
Hybrid M1/M2 macrophages			• CD11c^+^CD206^+^ [47] • F4/80^hi^CD11c^+^CD206^+^ [44]		ATMs phenotype isolated from obese mice [44].
Macrophages in human visceral adipose			• CD14^+^CD16^+^CD36^high^ [48] • CD14^+^CD16^−^CD163^+^		Proinflammatory macrophages Anti-inflammatory macrophages

Macrophages with different phenotypes perform diverse functions in adipose tissue. MMe macrophages are driven by high levels of glucose, insulin, and palmitate through the p62 and PPARγ pathways, with surface markers such as ABCA1, CD36 and PLIN2. MMe macrophages secrete cytokines such as IL-6 (NOX2-dependent), performing functions that remove dead adipocyte debris. CD9 macrophages are driven through the AP-1 subunit, JunB, NF-κB and subunit p65 pathways, possess the surface markers CD9, CD16 and CD206, and secrete cytokines such as IL-1α, IL-18 and TNF. Ly6c macrophages are driven through the CTCF pathway, with their cell surface markers CD11b and Ly6c. Ly6c macrophages perform functions that regulate the adipogenesis process. MFe^hi^ macrophages are driven by high iron, express CD163, Tfrc, Hmox1, ferritin light and heavy chains (Ftl1 and Fth1, respectively), ceruloplasmin (Cp) and ferroportin-1 (Slc40a1). The cell surface markers of antioxidant macrophages (Mox) are CX3CR1^neg^ and F4/80^lo^HO1^+^Txnrd1. They are predominant ATM phenotypes in lean adipose tissue and respond to oxidized phospholipids (OxPLs) by upregulating Nrf2-dependent antioxidant enzymes. The cell surface markers of hybrid M1/M2 macrophages are F4/80^hi^CD11c^+^CD206^+^. The cell surface markers of macrophages in human visceral adipose are CD14^+^CD16^+^CD163^high^ and CD14^+^CD16^−^CD163^+^

### Macrophages in a crown-like structure of adipose tissues

ATMs adopt a metabolically activated (MMe) phenotype to eliminate dead adipocytes in the way of lysosomal exocytosis [49]. In contrast to classically activated macrophages expressing cell surface markers such as CD38, CD319, and CD274, MMe macrophages specifically overexpress ABCA1, CD36, and PLIN2 regulated by p62 and PPARγ [37]. Recently, it has been revealed that MMe macrophages release IL-6 in an NADPH oxidase 2 (NOX2)-dependent manner, which signals through glycoprotein 130 (GP130) on triple-negative breast cancer (TNBC) cells to promote stem-like properties including tumor formation [38]. MMe macrophages exhibit a pleiotropic effect on tissue environmental homeostasis, which can cause corresponding pathophysiological changes to vary with the progression of obesity. NADPH oxidase 2 (NOX2) has been identified as a driver of the inflammatory and adipocyte-clearing properties of MMe macrophages. Nox2^−/−^ mice show mildly improved glucose tolerance in early diet-induced obesity (DIO) compared with wild-type mice due to decreased secretion of inflammatory factors [38]. However, when advanced to late DIO, inactivation of the lysosomal exocytosis function would result in tissue damage due to from severe lipid accumulation [38].

CD9^+^ ATMs, which are lipid-laden and localized to CLSs, are responsible for the inflammatory signature of obese adipose tissue, and adoptive transfer of CD9^+^ ATMs induces obese-associated inflammation in lean mice [40]. CD9^+^ ATMs express higher levels of the surface markers CD16 and CD206 than CD9^−^ ATMs and are enriched for transcription factors AP-1 and NF-κB with associated genes such as *Ccl2, Il1a, Il18,* and *Tnf* [40]. In contrast to CD9 ATMs with a signature of metabolic activation, Ly6c ATMs express genes related to angiogenesis and tissue organization. Ly6c ATMs provide normal adipose physiology upon adoptive transfer by inducing genes related to cholesterol and lipid biosynthesis [40].

Recently, a novel and conserved macrophage named lipid-associated macrophage (LAM) with high levels of the lipid receptor Trem2 has been proven to be the predominantly expanded immune cell subset in adipose tissue in multiple obesity-related mouse models [50]. The formation of LAM cells in CLS in adipose tissue is driven by Trem2 signaling, and knockout of Trem2 in bone marrow cells deteriorated the metabolic outcomes of obesity, suggesting that Trem2^+^ LAM cells are crucial for the prevention of metabolic disorders upon loss of adipose tissue homeostasis [50].

### Iron-rich macrophages in adipose tissues

A study describes a novel population of alternatively activated iron-rich ATMs named MFe^hi^, which display an anti-inflammatory and iron-recycling gene expression profile [42]. MFe^hi^ ATMs are capable of storing excess iron from dietary and intraperitoneal supplements mainly through MFe^lo^ ATM incorporation to expand the MFe^hi^ pool [43]. The impaired iron handling in MFe^hi^ ATMs has impacted iron distribution, causing adipocyte iron overload and AT dysfunction in obesity [42]. Compared with LFD-fed mice, HFD-feeding increased *Itgax, Ccr7, Tnfα* and *Il1β* expression and decreased M2 marker expression of *Stab1* and *Clec10a* in MFe^hi^ ATMs [42].

### Antioxidant macrophages in adipose tissues

Oxidized phospholipids (OxPLs) have been identified as endogenous danger associated molecular patterns (DAMPs) with characteristics of oxidative damage to tissues. Macrophages have the capacity to translate tissue oxidation status into either antioxidant or inflammatory responses by sensing OxPLs [46]. Antioxidant macrophages (Mox) respond to OxPLs by upregulating Nrf2-dependent antioxidant enzymes [45] and producing the antioxidant glutathione to suppress regular energy metabolism [46]. A unique population of CX3CR1^neg^/F4/80^low^ ATMs that resemble the Mox phenotype (Txnrd1^+^HO1^+^) has been demonstrated to be the predominant ATMs in lean adipose tissue [44].

### Macrophages in visceral adipose tissues and subcutaneous adipose tissues

Macrophage polarization in human visceral adipose tissue is related to fatty acid metabolism, cell membrane composition, and diet. CD11c^+^CD163^+^ ATMs have been confirmed to accumulate in both VAT and SAT of obese individuals and were found to be clearly correlated with body mass index and production of reactive oxygen species [27]. Proinflammatory and anti-inflammatory macrophages from human VAT have been determined by flow cytometry as CD14^+^CD16^+^CD36^high^ and CD14^+^CD16^−^CD163^+^, respectively [48]. Macrophages in obese adipose tissue are CD11c^+^CD206^+^, interpreted to be hybrid M1/M2 macrophages [47].

### Other adipose tissue macrophages

Macrophages exhibit correlations with adipocyte accumulation in human skeletal muscles. IL-1β-polarized macrophages (M(IL-1β)) drastically reduced fibroadipogenic progenitors (FAP) adipogenic potential, while IL-4-polarized macrophages (M(IL-4)) enhanced FAP adipogenesis [51]. Tissue-resident NRP1^+^ macrophages can drive healthy weight gain and maintain glucose tolerance. Ablation of NRP1 in macrophages compromised lipid uptake in these cells, which reduced substrates for fatty acid β-oxidation and shifted energy metabolism of these macrophages toward a more inflammatory glycolytic metabolism [52].

## Macrophages and adipocytes interact in physiological and pathological events

White adipose tissue serves as an energy-storage organ and plays a homeostatic role in energy dissipation [53]. Moreover, brown adipose tissue generates heat through uncoupled respiration, protecting against hypothermia, hyperglycemia and hyperlipidemia [54, 55]. In addition, beige adipocytes inducibly express mitochondrial uncoupling protein UCP1 in response to cold exposure and execute a thermogenic and energy-dissipating function interspersed within white adipose tissue [56].

### Macrophage-adipocyte interaction in energy metabolism

It has been reported that brown adipocytes release CXCL14 to promote adaptive thermogenesis via M2 macrophage recruitment, BAT activation and white fat browning [57]. Likewise, it has been identified that ATM-generated miR-10a-5p is a potential regulator of inflammation in ATMs and induces beige adipogenesis in adipocyte stem cells (ASCs) [58]. Currently, it has been delineated that alkylglycerol-type ether lipids (AKGs) such as breast milk-specific lipid species are metabolized by ATMs to platelet-activating factor (PAF), which ultimately activates IL-6/STAT3 signaling in adipocytes and triggers beige adipose tissue development in infants [59]. In contrast, the partial depletion of CD206^+^ M2 macrophages elevates the number of beige progenitors in response to cold in genetically engineered CD206DTR mice [60]. M1 macrophages may be partially associated with failure in perigonadal WAT that undergoes browning, as evidenced by removal of macrophages enhancing cold-induced UCP1 expression [61].

Additionally, inflammatory macrophages adhere to adipocytes, mediated by α4 integrin binding to VCAM-1, inhibiting thermogenic UCP1 expression in an Erk-dependent way, thereby impairing beige adipogenesis in obesity [62]. Furthermore, macrophages modulate energy metabolism of WAT in an activation-dependent paracrine way, as evidenced by how CD163^high^CD40^low^ macrophages activated by IL-10/TGF-β downregulated the expression of mitochondrial complex III (UQCRC2) gene/protein and ATP-linked respiration, whereas CD40^high^CD163^low^ macrophages activated by LPS/IFN-γ potentiated adipocyte mitochondrial activity [63].

In addition, JAK2, a key mediator downstream of various cytokines and growth factors, which is deficient in macrophages, improves systemic insulin sensitivity and reduces inflammation in VAT and liver in response to metabolic stress [64]. The nuclear lamina is a protein network structure surrounding the nuclear material that participates in a number of intranuclear reactions. Lamin A/C mediates ATM inflammation by activating NF-κB to promote proinflammatory gene expression, hence hastening obesity-associated insulin resistance [65].

### Macrophage-adipocyte interaction in glycolysis and OXPHOS

Growing evidence has shown that ATMs adopt a unique metabolic profile such as glycolysis and oxidative phosphorylation (OXPHOS), while fatty acid oxidation, glycolysis and glutaminolysis have been reported to facilitate ATMs to release cytokine in lean adipose tissue [66]. Inflammatory macrophages (M1) have metabolic features such as increased succinate-driven Hif1α-dependent glycolysis [66] and reduced phosphorylation, as well as a TCA cycle break-point at *Idh* [67]. On the other hand, anti-inflammatory macrophages (M2) possess characteristics such as enhanced OXPHOS, UDP-GlcNAc biosynthesis and glutamine-related pathway flows [67]. Cpt2^A−/−^ mice in which mitochondrial long chain fatty acid β-oxidation was deleted were induced to undergo loss of BAT and a reduction in UCP1 expression by administration of β3-adrenergic (CL-316243) or thyroid hormone (GC-1) agonists, suggesting that adipose fatty acid oxidation is required for the development of BAT during both activation and quiescence [68].

Release of succinate by adipose tissue is a response to hypoxia and hyperglycemia. Succinate receptor 1 (SUCNR1) activation mediates macrophage infiltration and inflammation in obesity, as evidenced by how Sucnr1^−/−^ mice displayed decreased macrophage numbers and increased glucose tolerance [69]. Adipose tissue hypoxia impact on preadipocytes and ATMs in obesity has been reviewed in detail in reference [70].

### Macrophages, adipocytes and nervous system

The interplay between neuroimmunology and immunometabolism is prevalent within adipose tissue, where immune cells and the sympathetic nervous system play a critical role in metabolic homeostasis and obesity [71]. The interaction between neurons and macrophages has influenced adipocyte biology and whole-body metabolism [72]. Although alternatively activated macrophages do not synthesize relevant amounts of catecholamines [73], a recent study has shown that Irs2^LyzM−/−^ mice are resistant to obesity upon HFD-feeding via regulation of sympathetic nerve function and catecholamine availability in adipose tissue to activate BAT and beigeing of WAT [74]. Macrophages deficient in Irs2 express an anti-inflammatory profile and catecholamine scavenging associated genes to support adipose tissue sympathetic innervation [74].

It has been supposed that neuron-associated macrophages (SAMs) pathologically accumulate in sympathetic nervous system (SNS) nerves of obese subjects in an organ-specific manner, acting as a norepinephrine (NE) sink and exerting proinflammatory activity [75]. Deletion of Mecp2 in CX3CR1^+^ macrophages impeded BAT sympathetic innervation, disrupting NE signaling required for expression of uncoupling protein 1 (UCP1) and BAT thermogenesis [76]. The impairment of catecholamine-induced lipolysis in aging was reversed by alteration of the expression of NLRP3, growth differentiation factor-3(GDF3) and monoamine oxidase A (MAOA) in AT macrophages via regulating the bioavailability of noradrenaline [77].

### Macrophage-adipocyte interactions in other aspects

The adipose tissue microenvironment interrupts late autophagosome maturation in macrophages, supporting enhanced lipid-droplet (LD) biogenesis and AT foam cell (FC) formation, thereby contributing to AT dysfunction in obesity [78]. Growth/differentiation factor 3 (GDF3) is an activin receptor-like kinase 7 (ALK7) ligand produced from CD11c^+^ macrophages to control lipolysis and direct ALK7-dependent accumulation of fat in vivo. It has been clarified that the GDF3-ALK7 axis between macrophages and adipocytes is tied to insulin regulation of both fat metabolism and mass [79]. Antigen presentation by either ATMs or adipocytes must be preserved in order to improve systemic glucose metabolism in HFD-fed mice [80]. Specific loss of APC function in ATMs yields mice that are more glucose tolerant. APC function loss in either ATMs or adipocytes, but not both, improves systemic glucose metabolism [80].

## Conclusion

ATMs responsible for immune surveillance in adipose tissue during HFD-induced obesity are reprogrammed to produce inflammatory and metabolic activated subsets. In addition to M1 and M2 subsets, ATMs with a variety of cell phenotypes to perform their roles in clearance of cellular debris, lipid metabolism, iron storage and energy metabolism in both physiological and pathological states. In summary, the current understanding of the characteristics of the biology and properties of macrophages in adipose tissues facilitates the elucidation of ATM polarization, metabolism and regulatory mechanisms. Fully exploration of ATMs functions in obesity can provide potential pharmacologic control points to prevent and treat obesity-related metabolic disorders. Furthermore, the microenvironment of adipose tissues in obesity needs further investigation, especially the epigenetic and transcriptional regulation of the physiological changes of adipocytes from the interplay between ATMs and adipocytes.

## Data Availability

Not applicable.

## Abbreviations

ATMs: Adipose tissue macrophages
AT: Adipose tissue
ADM: Adipocyte-derived microvesicles
ADSCs: Adipose-derived stem cells
ASCs: Adipocyte stem cells
AKGs: Alkylglycerol-type ether lipids
ALK7: Activin receptor-like kinase 7
AdExos: Adipocytes release lipid-laden exosomes
BAT: Brown adipose tissue
CB1: Cannabinoid receptor 1
CHOP: C/EBP homologous protein
CLS: Crown-like structure
DAT: deep Adipose tissue
DAMPs: Danger associated molecular patterns
DIO: diet-Induced obesity
ER: Endoplasmic reticulum
FNDC5: Fibronectin type III domain-containing protein 5
FGF21: Fibroblast growth factor 21
FAP: Fibro-adipogenic progenitors
FC: Foam cells
GP130: Glycoprotein 130
GDF3: Growth/differentiation factor 3
IRE1α: Inositol-requiring enzyme 1α
Klf4: Krüppel-like factor 4
IL-1β: Interleukin-1β
LAM: Lipid-associated macrophage
LDs: Lipid-droplets
MCP-1: Monocyte chemotactic protein 1
Mox ATMs: Antioxidant macrophages
Mme: Metabolically activated phenotype
M1: Classically activated macrophages
M2: Alternatively activated macrophages
NFAT c3: NUCLEAR factors of activated T cells c3
NPFF: Neuropeptide FF
NOX2: NADPH oxidase 2
NE: Norepinephrine
OxPLs: Oxidized phospholipids
OXPHOS: Oxidative phosphorylation
PAF: Platelet-activating factor
SAT: Subcutaneous adipose tissue
SGLT: Sodium-glucose cotransporter
SUCNR1: Succinate receptor 1
SAMs: Neuron-associated macrophages
SNS: Sympathetic nervous system
TAG: Triacylglyceride
TNBC: Triple-negative breast cancer
UCP1: Uncoupling protein 1
UPRs: Unfolded protein reactions
VAT: Visceral adipose tissue
WAT: White adipose tissue
